# A Case of Hydrocele Cured by the Injection of Tinct. of Iodine

**Published:** 1862-03

**Authors:** J. N. Green

**Affiliations:** Stilesville, Indiana


### A CASE OF HYDROCELE CURED BY THE INJECTION OF TINCT. OF IODINE.

By J. N. GREEN, M. D.,

*Of Stilesville, Indiana.*

A few months since, I was consulted by H., aged about 25, who had suffered for several years previous with an hydrocele. He had, as he informed me, the thing "tapped" repeatedly by Dr. Mathews, with no other result than temporary relief from the swelling, the effusion returning in a short time, and requiring the operation to be repeated again.

Becoming justly tired of this method of treatment, the patient was induced to inquire after a radical cure, if such could be found. I accordingly recommended to him with much confidence the injection of tinct. of Iodine into the sac as practiced by Prof. Brainard. He at once consented to the operation; and I introduced a trocar into the sac, drawing off the effused fluid and injected what I supposed to be a sufficient amount of tinct. of Iodine in its stead. This done, I told the patient it might be necessary to repeat the process once or twice, but assured him if persisted in he might expect an entire cure. He expressed his willingness to submit to the requisite number of operations to secure this desirable consummation. But fortunately, a repetition of the operation was unnecessary; the patient was cured by the first injection, thanks to Dr. Brainard and the tinct. of Iodine.

I do not report this case because there is anything remarkable in it, or skill in the operation; but simply to add the weight of this case to the large and daily increasing amount of evidence before the medical public, of the usefulness of iodine in the cure of this and analogous diseases. If a case of several years standing may be thoroughly cured by a single injection, what a source of relief is held out to the thousands in the country, afflicted with this unpleasant disease. I trust, furthermore, that the attention of the medical profession may be directed to this matter, and that they will treat their cases of hydrocele as above.

